# Falling fowl of the chicken reference genome: pitfalls of studying polymorphic endogenous retroviruses

**DOI:** 10.1186/s12977-021-00555-3

**Published:** 2021-04-20

**Authors:** Andrew S. Mason

**Affiliations:** Jack Birch Unit for Molecular Carcinogenesis, The Department of Biology and York Biomedical Research Institute, The University of York, York, YO10 5DD UK

**Keywords:** Endogenous retrovirus, ALVE, Chicken, Avian leukosis virus, Reference genome, Polymorphic ERVs

## Abstract

High quality reference genomes have facilitated the study of endogenous retroviruses (ERVs). However, there are an increasing number of published works which assume the ERVs in reference genomes are universal; even those of evolutionarily recent integrations. Consequently, these studies fail to properly characterise polymorphic ERVs, and even propose biological functions for ERVs that may not actually be present in the genomes of interest. Here, I outline the pitfalls of three studies of chicken endogenous Avian Leukosis Viruses (ALVEs or “ev genes”: the “original” ERVs), all confounded by the assumption that the reference genome provides a representative ALVE baseline.

## Main text

The recently concluded collection on “Endogenous Retroviruses in Evolution and Disease”, shared between this journal and *Mobile DNA*, has highlighted the impact of high-throughput sequencing for ERV annotation and characterisation. High-quality reference genomes have been a treasure trove for ERV discovery, and this will only continue with the rapidly progressing Vertebrate Genomes Project [1]. However, each reference genome offers only a snapshot of ERV diversity. The interpretation of polymorphic ERVs remains an outstanding challenge, particularly with a reliance on short read sequencing technologies which cannot uniquely distinguish between recently-integrated, intact ERVs with few, likely undescribed, discriminating variants. Furthermore, reference genomes are commonly considered, incorrectly, to be representative of that species’ genomic diversity. Consequently, several recent studies of ALVEs in chicken have generated highly interesting data, yet present misinterpreted conclusions.

ALVEs were the first ERVs to be described following efforts to control exogenous ALV in commercial flocks [2]. ALVEs exhibit the canonical retroviral structure without accessory genes, and have shorter long terminal repeats (LTRs) than exogenous ALVs, rendering them slow-transformers [3]. ALVEs remain of interest to both industry and academia for their impact on poultry characteristics [4, 5], historical negative associations with productivity traits [6, 7], and the complex interactions with exogenous viruses, including ALV [8, 9] and non-retroviruses, such as Marek’s Disease virus (MDV) [10]. The current chicken reference genome (GRCg6a), notably derived from a modern red junglefowl (the pre-domesticated ancestor of chickens), contains two ALVEs [11]. The structurally-intact ALVE-JFevB is, so far, unique to the reference genome individual. Conversely, the highly expressed ALVE6 (indicating its order of discovery in White Leghorn chickens in the 1980s) is truncated to just the *envelope* and 3′LTR, and is widespread, yet polymorphic, among commercial layers and broilers, but has not been identified in other red junglefowl. Excluding the low numbers observed in highly-selected Leghorns, ALVE abundance is typically in excess of six integrations per genome, and is usually > 10 in non-commercial lines and available red junglefowl datasets [12, 13]. Previous work has shown that despite the morphological and behavioural characteristics of the reference individual [14], this genome is heavily introgressed with the White Leghorn breed and not representative of wild red junglefowl, modern or ancestral [15].

The aim of these points is not to dismiss or diminish the importance of reference genomes, but rather to make clear that the chicken reference genome does not provide a baseline for: (1) specific ALVE integrations in chickens; (2) the typical ALVE abundance of chicken genomes; or (3) the pre-domestication ALVE state in the red junglefowl genome. Intuitively, you need to know what ALVEs your chicken has before suggesting what those ALVEs might be doing.

In their 2017 study, Hu and colleagues [16] studied heterogeneity in ALVE expression across tissues at two ages, suggesting a role in innate immunity based on high and sustained expression in lung and spleen. Using cell lines, they then observed reductions in ALVE expression when cells were infected with the retroviruses ALVJ and reticuloendotheliosis virus, but increased expression when infected with the herpesvirus MDV, particularly of ALVE *envelope* transcripts. When viruses are so commonly studied in isolation, this work showing modified expression during effective co-infection is of particular interest, especially given recent work on MDV vaccination and elevated incidence of spontaneous lymphoid-like tumours [10]. However, the authors attribute all ALVE expression specifically to ALVE1, without confirming its presence in the genome. In fairness, all birds and cell lines used in this study were derived from White Leghorns, where ALVE1 is highly prevalent yet still polymorphic, even within individual flocks [12]. Furthermore, most White Leghorns contain 3 or more ALVE integrations, and the common ALVE3, ALVE6 and ALVE9 elements all exhibit high *envelope* expression. In isolation, this ALVE1 assumption could be seen as an oversight based on its prevalence in White Leghorn flocks.

In a 2019 study the same group reported an antisense long non-coding RNA specifically derived from ALVE1 (lnc-ALVE1-AS1), which they showed to induce antiviral innate immunity consistent with a type I interferon response [17]. Again, these data are interesting, particularly as overexpression of lnc-ALVE1-AS1 was shown to significantly reduce ALVJ titre. However, the lnc-ALVE1-AS1 schematic in their Fig. 3B incorrectly identifies the assembled, reference-genome-specific ALVE-JFevB as ALVE1, suggesting that the authors were unaware of ALVE1 polymorphisms, or the presence of other ALVEs, in either study.

In both papers [16, 17], the broad results remain interesting, but the nuance and translation of the work is hindered by not identifying which ALVE, or combination, is responsible. A final, more problematic example is that of Sun and colleagues [18], who presented an otherwise exciting paper about the genesis of PIWI-interacting RNA (piRNA) defence against ALVEs, a novel finding as piRNAs had not previously been shown to suppress any competent infectious virus. Sun and colleagues worked largely with White Leghorn data, but also utilised expression data from red junglefowl, although not the same individual, or population, as the reference. Whilst the authors did identify ALVE6 in their White Leghorns, and check for other known ALVE integrations, they did not do the same in the red junglefowl. Rather, the authors assumed the ALVE complement of the reference was representative of the pre-domesticated state, even after saying they could not discount lineage-specific indels with other transposable elements. Consequently, the authors hypothesised a domestication-associated harnessing of ALVE6 for piRNA, and related this to its modulatory effect on ALV infection, long-recognised as receptor interference [9]. Comparative studies of piRNA between breeds (of known ALVE status), as suggested by the authors, are crucial to truly elucidate the role of ALVE6, or other ALVEs, in piRNA-mediated defence.

In each of these three case studies, comprehensive ALVE identification would have aided interpretation. Fortunately, as high-throughput sequencing approaches have become more accurate and cost-effective, this has become more achievable. ALVE integrations can be detected confidently from whole genome sequencing data [12], utilising enrichment approaches to exclusively assess study population ALVE diversity if budgets require [19]. Furthermore, these approaches are broadly applicable to ERVs across vertebrate genomes. Functional ERV annotation is itself a different and challenging matter, often due to high sequence homology, now being addressed in part by long-read technologies. However, until the utility and scope of pan-genome analysis matures, we still heavily depend on single individual reference genomes to interpret polymorphic ERVs. We just need to ensure that this dependence does not preclude robust conclusions.

## Data Availability

Not applicable.

## Abbreviations

ALV: Avian leukosis virus
ALVE: Avian leukosis virus subgroup E
ALVJ: Avian leukosis virus subgroup J
ERV: Endogenous retrovirus
LTR: Long terminal repeat
MDV: Marek’s disease virus
piRNA: PIWI-interacting RNA
